# Plasma Exosomes Associated with Growth Divergence in High-Density Cultured Grass Carp (*Ctenopharyngodon idella*): miRNA-Protein Profiling Reveals Cross-Tissue Communication Networks

**DOI:** 10.3390/ijms27136059

**Published:** 2026-07-06

**Authors:** Tengfei Zhu, Zhipeng Zheng, Hao Chen, Yingying Yu, Huayang Guo, Baosuo Liu, Kecheng Zhu, Nan Zhang, Lin Xian, Shuhui Zheng, Yang Liu, Songlin Chen, Dianchang Zhang

**Affiliations:** 1 Key Laboratory of South China Sea Fishery Resources Exploitation and Utilization, Ministry of Agriculture and Rural Affairs, South China Sea Fisheries Research Institute, Chinese Academy of Fishery Sciences, Guangzhou 510300, China; tengfei.zhu@outlook.com (T.Z.); 18475354895@163.com (Z.Z.); hchen00@foxmail.com (H.C.); guohuayang198768@163.com (H.G.); liubaosuo343@163.com (B.L.); zkc537@163.com (K.Z.); 398730316@163.com (N.Z.); xlinxlxl@163.com (L.X.); zsh0770711@163.com (S.Z.); 2 Sanya Tropical Fisheries Research Institute, Sanya 572018, China; 3 Guangdong Provincial Key Laboratory of Animal Molecular Design and Precise Breeding, School of Animal Science and Technology, Foshan University, Foshan 528225, China; yuyy999@yeah.net; 4 Guangdong Engineering Research Center of Key Technologies and Equipment R&D on Modern Marine Ranching, Guangzhou 510300, China; 5 Guangdong Provincial Engineer Technology Research Center of Marine Biological Seed Industry, Guangzhou 510300, China; 6 State Key Laboratory of Mariculture Biobreeding and Sustainable Goods, Yellow Sea Fisheries Research Institute, Chinese Academy of Fishery Sciences, Qingdao 266000, China; liuyang@ysfri.ac.cn (Y.L.); chensl@ysfri.ac.cn (S.C.)

**Keywords:** plasma exosomes, *Ctenopharyngodon idella*, cross-tissue communication, microRNA, proteomics, growth performance, high-density aquaculture, biomarkers

## Abstract

Grass carp (*Ctenopharyngodon idella*) is a major freshwater aquaculture species in China, but its growth is limited under intensive high-density farming. This study aimed to investigate the characteristics of plasma exosomes associated with distinct growth performance by isolating and characterizing exosomes from fast- and slow-growing grass carp after nine months of culture. Exosomes showed typical morphology and expressed characteristic markers (CD63, CD81, TSG101). Small RNA sequencing identified 3325 miRNAs, with 177 highly abundant miRNAs differentially expressed: immune-related miRNAs were upregulated, while development-inhibitory miRNAs were downregulated in fast-growing fish. Target gene enrichment highlighted pathways in neural and skeletal development and amino acid metabolism. Integrative analysis across tissues revealed 26 miRNAs with coordinated expression patterns between plasma exosomes and brain, liver, or muscle, validated by qPCR. DIA proteomics quantified 4203 proteins, identifying 843 differentially enriched proteins linked to immune response, energy metabolism, and endoplasmic reticulum stress. Notably, TYMP was upregulated in muscle and exosomes, while several proteins (e.g., GYG2, BHMT) showed coordinated downregulation across tissues and exosomes in large fish. These results provide comprehensive evidence of exosome-mediated cross-tissue communication in teleosts and suggest a potential role for plasma exosomal miRNAs and proteins as non-invasive biomarkers correlated with growth status in aquaculture.

## 1. Introduction

Grass carp (*Ctenopharyngodon idella*) is one of the most important cultured freshwater fish species in China, with an annual production of 5.94 million tons in 2023, accounting for 17.4% of the country’s total freshwater aquaculture output [1]. Under intensive aquaculture conditions, high-density stocking is commonly practiced to maximize production efficiency. However, this practice often induces chronic stress, leading to substantial growth variation among individuals and increased mortality rates [2]. The breeding cycle for grass carp typically requires 3–4 years, which significantly limits the efficiency of genetic improvement programs. Because growth divergence under chronic crowding stress is a complex, systemic phenomenon involving multiple physiological pathways, understanding the underlying mechanisms requires a holistic investigation into how distant tissues regulating growth coordinate their metabolic and adaptive responses. In this context, effective inter-organ communication mediators are essential for coordinating these systemic physiological adaptations.

Exosomes are small extracellular vesicles (30–200 nm in diameter) with a characteristic lipid bilayer structure that are secreted by nearly all cell types [3]. These nano-sized vesicles encapsulate a complex cargo of proteins, lipids, and nucleic acids, including microRNAs (miRNAs), and serve as crucial mediators of intercellular and interorgan communication [4]. Once released into the extracellular space, exosomes can be transported through body fluids such as blood plasma and cerebrospinal fluid, subsequently internalized by recipient cells via membrane fusion or endocytosis [5]. Through this mechanism, exosomes facilitate the horizontal transfer of functional molecules between distant tissues, thereby regulating various physiological processes, including development, immune responses, and metabolic homeostasis [6,7]. In aquatic organisms, extracellular vesicles, particularly exosomes, serve as pivotal mediators of environmental stress adaptation, host–pathogen interactions, and reproductive regulation, underscoring their growing significance in intensive aquaculture research [8,9,10,11].

Among the various molecular cargoes carried by exosomes, miRNAs have attracted considerable attention due to their potent regulatory functions. miRNAs are small non-coding RNA molecules, typically 18–25 nucleotides in length, that regulate gene expression post-transcriptionally by binding to complementary sequences in the 3′ untranslated region of target mRNAs, leading to mRNA degradation or translational repression [12]. In teleost fish, miRNAs have been shown to participate in diverse developmental and physiological processes, including embryogenesis, organogenesis, immune regulation, and muscle development [13,14,15,16]. Encapsulation within exosomes protects circulating miRNAs from degradation, enabling them to function as stable biomarkers and endocrine signaling vectors that traverse the systemic circulation to modulate physiological responses in distant recipient organs [17,18,19].

Despite increasing recognition of the importance of exosome-mediated communication in vertebrate physiology, limited research has focused on exosome function in teleost fish, particularly in the context of growth regulation. Furthermore, the potential of plasma exosome-associated miRNAs and proteins as biomarkers for growth performance in aquaculture species remains largely unexplored. Understanding the molecular signatures distinguishing fast- and slow-growing individuals under high-density culture conditions could provide valuable insights into the mechanisms underlying growth variation and facilitate more efficient breeding programs.

In this study, we hypothesized that plasma exosomes from fast-growing and slow-growing grass carp raised under identical high-density conditions carry distinct miRNA and protein signatures that reflect different physiological states. Furthermore, we proposed that these exosomal cargoes mediate cross-tissue communication between growth-related organs (brain, liver, and muscle), thereby influencing overall growth performance. To test this hypothesis, we conducted integrated small RNA sequencing and data-independent acquisition (DIA) mass spectrometry analyses of plasma exosomes from large (fast-growing) and small (slow-growing) grass carp individuals. The primary objectives of this study were to: (1) characterize the miRNA and protein profiles of plasma exosomes from grass carp with distinct growth performance; (2) identify differentially expressed miRNAs and proteins that may serve as biomarkers for growth status; and (3) investigate the potential associations between exosome profiles and cross-tissue communication networks under high-density aquaculture conditions. It should be noted that the present study focuses on identifying molecular correlations, and whether these distinct signatures are the causes or the consequences of growth divergence remains to be elucidated through future functional validations.

## 2. Results

### 2.1. Growth Performance

**Figure 1:**
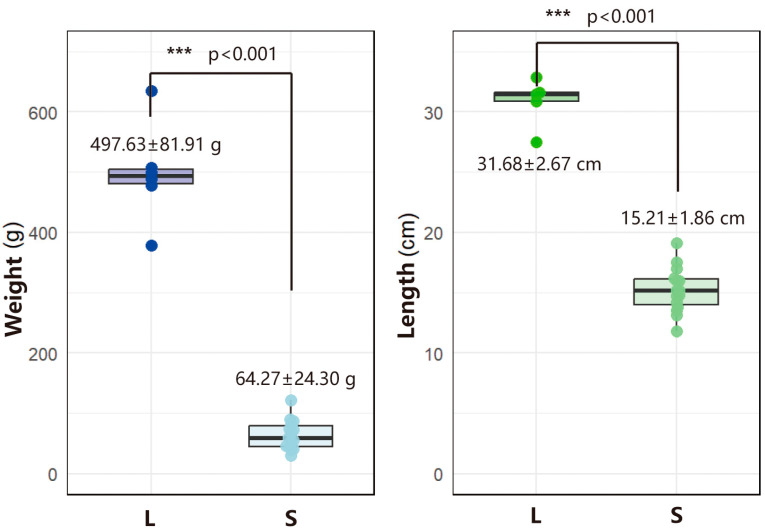
Growth performance of grass carp under high-density aquaculture conditions. Comparison of body weight and length between large (L, *n* = 6) and small (S, *n* = 15) grass carp individuals after nine months of culture in a high-density pond. Data are presented as mean ± standard deviation (SD). *** *p* < 0.001 determined by unpaired Student’s *t*-test. The significant differences in both body weight and length between the two phenotypic groups provide a basis for subsequent analysis of plasma exosomal differences.

After a nine-month period of cultivation in a high-density pond, grass carp exhibiting significant variations in body weight and length were collected for analysis. The cohort of six larger fish (L group) demonstrated an average body weight of 497.63 ± 81.91 g and an average body length of 31.68 ± 2.67 cm. In contrast, the group of fifteen smaller fish (S group) exhibited an average body weight of 64.27 ± 24.30 g and an average body length of 15.21 ± 1.86 cm. Statistical analysis revealed that both the body weight and length of the larger fish were significantly greater than those of the smaller fish, indicating a pronounced disparity in growth between the two groups (Figure 1). These findings establish a foundational framework for subsequent investigations into the differential molecular cargo of plasma-derived exosomes.

### 2.2. Plasma Exosome Characterization

**Figure 2:**
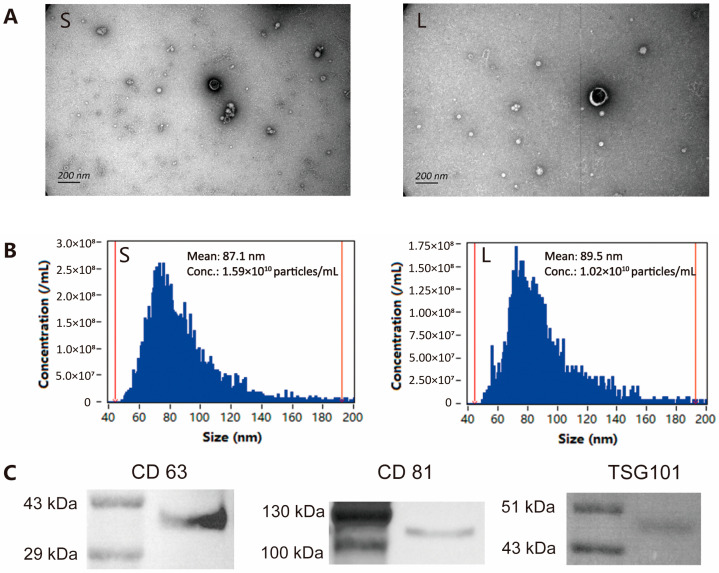
Characterization of plasma exosomes from grass carp. (**A**) Transmission electron microscopy (TEM) images of isolated plasma exosomes from grass carp. The exosomes exhibit typical cup-shaped morphology with a concave appearance, characteristic of exosomal vesicles. Scale bars: 200 nm. (**B**) Nano-flow cytometry (nFCM) analysis of particle size distribution and concentration. Left panel: Particle size distribution profiles of exosomes from S group and L group, showing that exosomes are predominantly distributed within the 50–190 nm range. The average particle size was 87.1 nm for S group and 89.5 nm for L group, with particle concentrations of 1.59 × 10^10^ particles/mL and 1.02 × 10^10^ particles/mL, respectively. (**C**) Western blot detection of exosomal marker proteins. CD63, CD81 and TSG101 were positively expressed in the plasma exosome samples.

Following the isolation of exosomes, we conducted further characterization of the extracted exosomes with respect to morphology, particle size, and marker proteins. Transmission electron microscopy (TEM) revealed a spherical morphology with a concave appearance, characteristic of the typical cup-shaped feature of exosomes (Figure 2A). Nanoflow particle size analysis indicated that these vesicles were predominantly distributed within the 50–190 nm range, with the average particle sizes of the L and S group vesicles being similar—89.5 nm and 87.1 nm, respectively—consistent with exosomal characteristics. Additionally, the particle concentrations obtained were 1.02 × 10^10^ particles/mL and 1.59 × 10^10^ particles/mL, suggesting that the yield of isolated vesicles falls within the normal range (Figure 2B). To conserve sample consumption, we combined exosome samples from groups L and S prior to protein extraction and subsequently identified exosome marker proteins via Western blot (WB) analysis. The results demonstrated positive expression of CD63, CD81, and TSG101 in the samples, with their bands appearing at approximately 29–43 kDa (CD63), 100–130 kDa (CD81), and 43–51 kDa (TSG101), respectively, aligning with their expected molecular weights (Figure 2C). These findings confirm the successful isolation of plasma exosomes using the classical ultracentrifugation method, with the total yield and concentration meeting the requirements for subsequent sequencing analysis.

### 2.3. Plasma Exosomal miRNA Sequencing and Profiling in Large and Small Fish Groups

To examine the variations in plasma exosomal miRNA expression profiles between the large and small fish groups, we performed plasma exosomal miRNA sequencing on three samples from each group. A total of 199.97 million raw reads were obtained, with individual sample counts ranging from 27.92 million to 41.32 million, with an average of 33.33 million. Following quality control and data filtering, 149.00 million high-quality clean reads were acquired, with sample counts ranging from 19.06 million to 34.16 million and an average of 24.83 million reads. The effective data rate, represented by the proportion of clean reads to raw reads, ranged from 68.25% to 82.67%, with an average value of 74.07%. These findings suggest that the sequencing data are of high quality and appropriate for subsequent miRNA identification and differential expression analyses. The analysis identified a total of 3325 miRNAs, comprising 2925 known miRNAs and 400 novel miRNAs. The number of miRNAs identified per sample ranged from 2278 to 2763. Specifically, the large fish group (L1–L3) exhibited 2433, 2278, and 2371 miRNAs, respectively, while the small fish group (S1–S3) exhibited 2532, 2763, and 2734 miRNAs, respectively. Within each sample, known miRNAs constituted 87.09% to 88.78%, whereas novel miRNAs constituted 11.22% to 12.91%. These preliminary sequencing metrics confirm reliable data output for the subsequent comparative and differential expression analysis (Table S1).

### 2.4. Identification of miRNAs Differentially Expressed in Plasma Exosomes and Functional Enrichment Analysis of Their Target Genes

**Figure 3:**
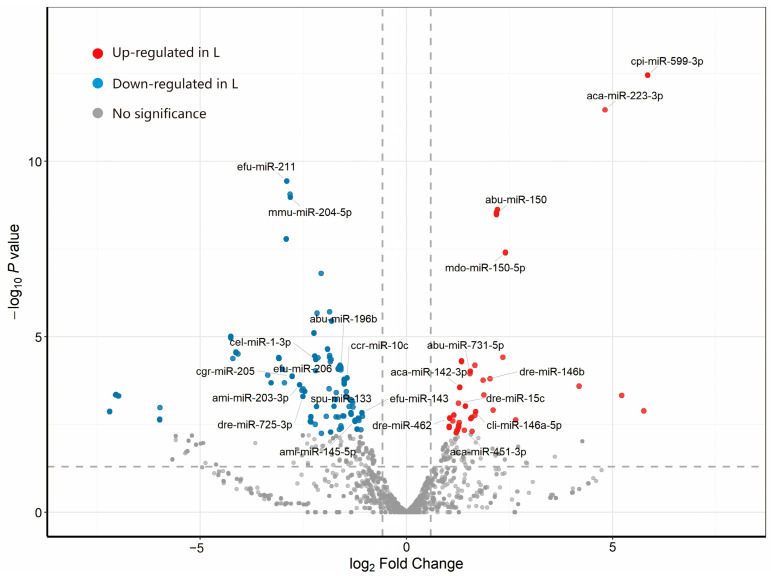
Identification of differentially expressed miRNAs in plasma exosomes between large and small grass carp. Volcano plot showing miRNA expression differences between L and S groups. Red dots represent significantly upregulated miRNAs (|FC| > 1.5, FDR ≤ 0.05) in large fish, blue dots represent significantly downregulated miRNAs (|FC| > 1.5, FDR ≤ 0.05) in large fish, and gray dots represent miRNAs without significant differential expression. The representative miRNAs are labeled. The vertical dashed lines indicate the threshold of a 1.5-fold change, and the horizontal dashed line indicates the statistical significance threshold of *p* < 0.05.

Following the application of edgeR filtering, a total of 2701 miRNAs were identified. Subsequent differential miRNA analysis was conducted between the large fish (L) and small fish (S) cohorts, employing |FC| ≥ 1.5, *p* Value ≤ 0.05, and FDR ≤ 0.05 as the selection criteria. This analysis resulted in the identification of 311 miRNAs exhibiting significant differential expression (Table S2). To minimize potential background noise from low-abundance transcripts, miRNAs were further filtered to retain only those with a normalized expression level exceeding 100 Transcripts Per Million (TPM) in at least one sample,, resulting in the selection of 177 miRNAs that demonstrated both significant differential expression and high expression in at least one sample. It was observed that the miR-142, miR-146, miR-150, miR-223, miR-462, miR-599, and miR-731 families were predominantly upregulated in large fish, whereas the miR-1, miR-10, miR-133, miR-143, miR-145, miR-196, miR-203, miR-204, miR-205, and miR-206 families were predominantly downregulated in small fish (Figure 3, Table S2).

**Figure 4:**
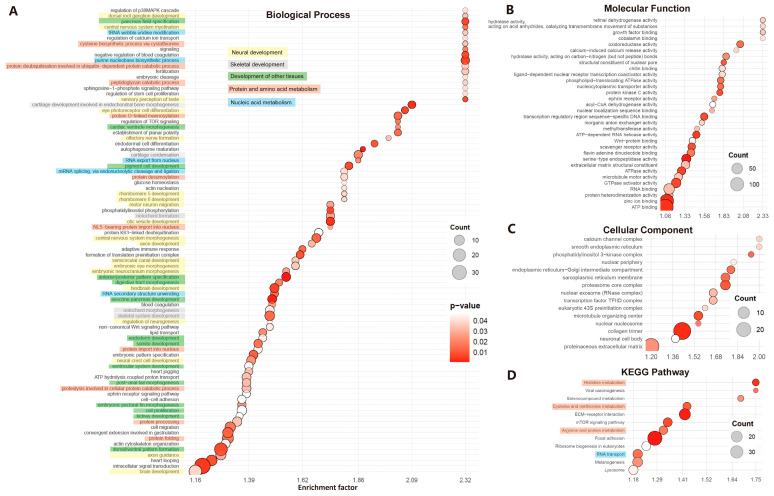
Functional enrichment analysis of target genes of differentially expressed plasma exosomal miRNAs between large and small grass carp. Biological process (**A**), molecular function (**B**) and cellular component (**C**) of GO and KEGG pathway (**D**) enrichment analysis of 2056 genes predicted to be targeted by 177 miRNAs with significant differences between large and small fish. The GO terms and KEGG pathway with *p*-value < 0.05 are shown. Circle size represents gene count; color indicates enrichment significance. Some important terms and pathways have been categorized: yellow highlights indicate neural development, gray highlights indicate skeletal development, green highlights indicate development of other tissues, red highlights indicate protein and amino acid metabolism, and blue highlights indicate nucleic acid metabolism.

To elucidate the potential biological processes associated with these 177 miRNAs in regulating growth in grass carp under high-density aquaculture conditions, the miRanda and RNAhybrid software were utilized to predict the target genes for these miRNAs (Table S3). GO and KEGG enrichment analyses of these 2056 target genes revealed significant enrichment in pathways associated with neural development (e.g., brain development, central nervous system morphogenesis, axon development, axon guidance), skeletal development (e.g., skeletal system development, cartilage condensation, notochord formation), protein and amino acid metabolism (e.g., cysteine biosynthetic process via cystathionine, histidine metabolism, cysteine and methionine metabolism, protein folding), nucleic acid metabolism (e.g., RNA export from nucleus, RNA secondary structure unwinding, RNA transport) (Figure 4).

### 2.5. miRNAs Potentially Delivered by Plasma Exosomes for Inter-Tissue Communication

Since exosomes are considered mediators of molecular information exchange between cells and tissues, the differential miRNAs in plasma exosomes are very likely to contain important information underlying the growth differences in grass carp under high-density conditions. Therefore, we further investigated the expression patterns of these 177 miRNAs in key growth-related organs of grass carp, including the brain, liver, and muscle (data from our parallel study). These observations align with our parallel transcriptomic and proteomic investigations of the same experimental grass carp cohort, which revealed tissue-specific metabolic reprogramming differences in the brain, liver, and skeletal muscle between large and small groups (manuscript under review). Upon excluding miRNAs that exhibited inconsistent patterns between plasma exosomes and tissues, we identified that of the 177 miRNAs with differential expression in plasma exosomes, 143 were also present in brain tissue. Notably, only dre-miR-146b exhibited a significant difference between large and small fish (*p* < 0.05). In the liver, 146 miRNAs were detected, with 23 demonstrating significant differences between large and small fish, predominantly from the miR-133 and miR-143 families (*p* < 0.05). Among these, three miRNAs (cli-miR-133c-3p, gga-miR-133c-3p, and pbv-miR-133c-3p) exhibited highly significant differences (*p* < 0.01). In muscle, 163 miRNAs were detected, with only two miRNAs (sup-miR-133 and xtr-miR-133) showing significant differences between large and small fish (*p* < 0.05) (Table S4). Consequently, these 26 miRNAs represent the coordinated regulatory relationship between plasma exosomes and tissues. This finding not only suggests a potential association between tissue miRNAs and plasma exosome miRNAs but also indicates that plasma exosome miRNAs could serve as non-invasive biomarkers for representing tissue miRNA information and growth regulatory information. We further extracted the expression data of the target genes of these 26 miRNAs from the transcriptomes of brain, liver, and muscle in both large and small grass carp. Among the 694 target genes examined across these three tissues, it was observed that, although there were 3 significantly different target genes in the brain, 11 in muscle, and 171 in the liver (FDR < 0.05), only 30 exhibited the inverse regulatory relationship consistent with miRNAs suppressing their target genes. Specifically, in large fish, dre-miR-146b was notably upregulated in both plasma exosomes and brain tissue, whereas its target genes, *rars2* and *ttc19*, were significantly downregulated in the liver transcriptome. Conversely, the miR-133 family exhibited significant downregulation in both plasma exosomes and the liver, while its target genes, including *cped1*, *alkbh5*, *saa*, *cbln14*, *egln1a*, *irf2bp2b*, and *wdr77*, were markedly upregulated in the muscle transcriptome. Additionally, efu-miR-143 and xtr-miR-133d were significantly downregulated in both plasma exosomes and muscle tissue, with their respective target genes, *wu: fb55g09*, *dennd2db*, *LOC127509884*, and *cped1*, *cbln14*, showing significant upregulation in muscle tissue (Table 1).

**Figure 5:**
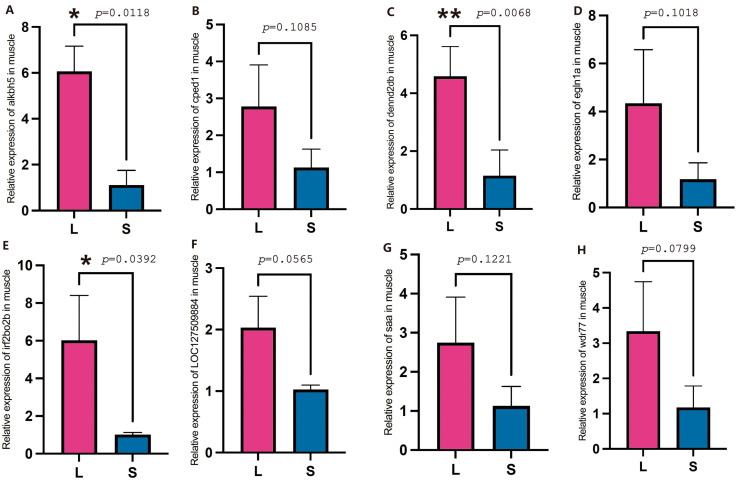
The relative expression of genes in muscle potentially targeted by plasma exosome miRNAs. The expressions of *alkbh5* (**A**), *cped1* (**B**), *dennd2db* (**C**), *egln1a* (**D**), *irf2bp2b* (**E**), *LOC127509884* (**F**), *ssa* (**G**) and *wdr77* (**H**) in muscle. Expression values were normalized by β-actin. Values are means (*n* = 3) with their standard deviations represented by vertical bars. (** *p* < 0.01; * *p* < 0.05 versus the controls, One-way analysis of variance, Tukey’s Test).

Upon further verification using qPCR of eight key genes in muscle tissue, it was observed that the expression levels of *alkbh5* (alkylation repair homolog protein 5), *dennd2db* (DENN/MADD domain containing 2Db), and *irf2bp2b* (interferon regulatory factor 2 binding protein 2b) were significantly elevated in the muscle tissue of larger fish. In parallel, the levels of bfl-miR-133, efu-miR-143, and pma-miR-133a, which target these genes, were found to be downregulated in the plasma exosomes and liver of the large fish. These synchronized, inverse expression profiles suggest a putatively coordinated regulatory network, wherein plasma exosomal miRNAs may potentially participate in modulating these muscle-specific transcripts, though direct functional causality remains to be mechanistically verified (Figure 5).

### 2.6. Plasma Exosomes Proteomic Profiling in Large and Small Fish Groups

In addition to miRNAs, exosomes also contain significant informational molecules such as proteins. Consequently, we conducted a high-resolution liquid chromatography-tandem mass spectrometry (LC-MS/MS) proteomic analysis of plasma exosomes from both large and small fish, utilizing the data-independent acquisition (DIA) mode. This analysis identified a total of 4203 proteins, with 3841 proteins detected in the large fish group and 4138 proteins in the small fish group. Among these, 3776 proteins were found in both groups, while 65 proteins were unique to the large fish group, and 362 proteins were exclusive to the small fish group. Following the exclusion of 202 proteins with substantial missing data (total valid value ≤ 1 in each group), effective data for 4001 proteins remained. Among these, 3737 proteins were identified in the large fish group and 3975 proteins in the small fish group, with 3711 proteins common to both groups, 26 proteins unique to the large fish group, and 264 proteins exclusive to the small fish group (Table S5).

### 2.7. Identification of Proteins Differentially Enriched in Plasma Exosomes and Their Functional Enrichment Analysis

**Figure 6:**
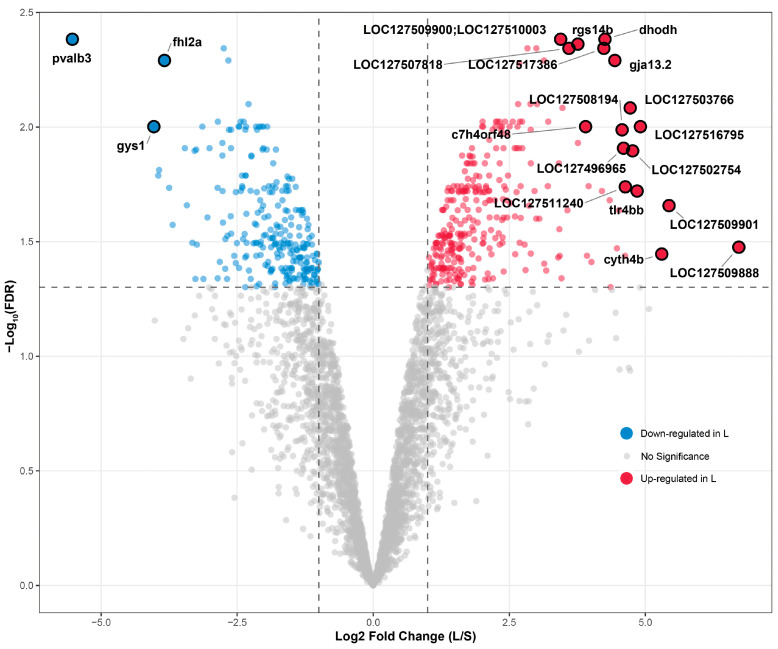
Volcano plot of differentially enriched proteins in plasma exosomes between large and small grass carp. The plot displays the 3711 proteins detected in both L and S groups. Red dots represent proteins significantly upregulated in large fish (|FC| > 2, FDR < 0.05), blue dots represent proteins significantly downregulated in large fish (|FC| > 2, FDR < 0.05), and gray dots represent proteins without significant differential expression. A total of 305 proteins were upregulated, and 248 proteins were downregulated in large fish. The top 20 significantly different proteins are labeled. The vertical dashed lines indicate the threshold of a 2-fold change, and the horizontal dashed line indicates the statistical significance threshold of *p* < 0.05.

Among the 3711 plasma exosome proteins identified in both the large and small fish groups, further analysis indicated that 305 proteins were significantly upregulated, while 248 proteins were significantly downregulated in the large fish group (FDR < 0.05, |FC| > 2) (Figure 6, Table S6).

**Figure 7:**
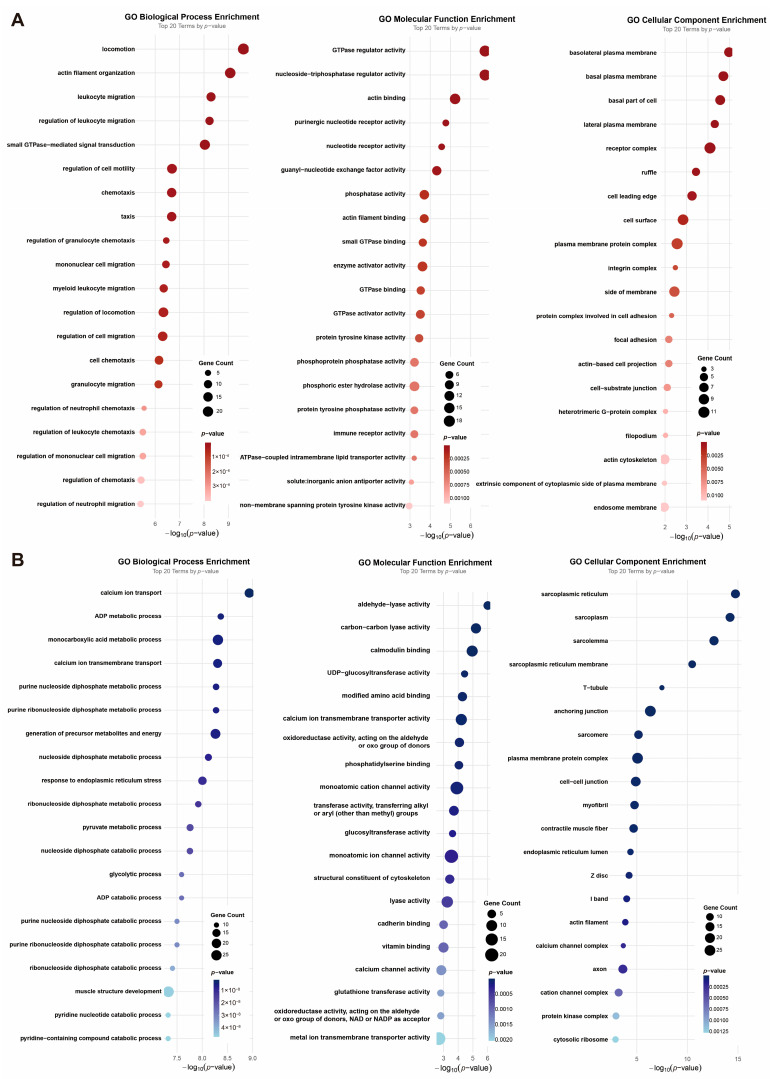
GO enrichment analysis of differentially enriched proteins in plasma exosomes. (**A**) GO enrichment analysis of proteins upregulated in plasma exosomes of large fish. The ternary plot systematically displays enriched terms across three GO categories: Biological Process (BP), Cellular Component (CC), and Molecular Function (MF). (**B**) GO enrichment analysis of proteins downregulated in plasma exosomes of large fish (or upregulated in small fish). Circle size indicates gene count, and color represents enrichment significance.

We integrated the 26 proteins detected exclusively in the large fish group with the 305 significantly upregulated proteins in the same group. Similarly, we combined the 264 proteins detected solely in the small fish group with the 248 significantly downregulated proteins in the large fish group, followed by a GO enrichment analysis. The analysis revealed that proteins significantly upregulated in large fish plasma exosomes were predominantly enriched in pathways related to immune cell migration, inflammatory response, cytoskeleton remodeling, and signal transduction. Key biological processes included locomotion, actin filament organization, leukocyte migration, chemotaxis, regulation of cell motility, and small GTPase-mediated signal transduction. The cellular components were enriched in the basolateral/lateral plasma membrane, receptor complex, integrin complex, and cell leading edge, while molecular functions were enriched in GTPase regulator activity, actin binding, guanyl-nucleotide exchange factor activity, and protein tyrosine kinase activity (Figure 7A). Conversely, proteins significantly downregulated in large fish plasma exosomes (or significantly upregulated in small fish plasma exosomes) were primarily enriched in processes such as energy metabolism, calcium ion regulation, muscle contraction, glycolysis, endoplasmic reticulum stress, and muscle structure development. Representative biological processes included calcium ion transport, glycolytic process, ADP metabolic process, response to ER stress, and muscle structure development. The cellular components were enriched in the sarcoplasmic reticulum, sarcolemma, T-tubule, sarcomere, and Z disk, and molecular functions were enriched in calmodulin binding, calcium channel activity, aldehyde-lyase activity, and cadherin binding (Figure 7B).

### 2.8. Proteins Potentially Delivered by Plasma Exosomes for Inter-Tissue Communication

**Figure 8:**
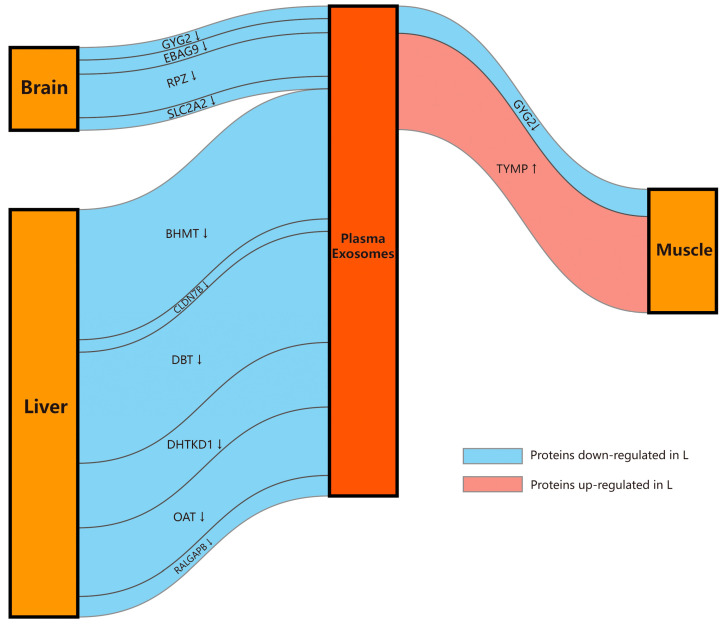
Hypothetical model of coordinated protein expression profiles across growth-related tissues and plasma exosomes in Grass Carp. Schematic diagram illustrating the coordinated expression patterns of proteins across growth-related tissues and plasma exosomes in large (L, fast-growing) and small (S, slow-growing) grass carp. The brain is positioned at the top, liver at the bottom, muscle on the right, and plasma exosomes in the center of the schematic. Red upward arrows with red shading represent proteins significantly upregulated in large fish (TYMP in muscle and plasma exosomes), while blue downward arrows with blue shading represent proteins significantly downregulated in large fish (all other proteins). Proteins showing coordinated downregulation in both tissues and plasma exosomes of large fish include: GYG2 (glycogenin-2) in brain, muscle, and plasma exosomes; EBAG9 (estrogen receptor binding site associated antigen 9), RPZ (rapunzel), and SLC2A2 (solute carrier family 2 member 2) in brain and plasma exosomes; and BHMT (betaine-homocysteine S-methyltransferase), CLDN7B (claudin 7b), DBT (dihydrolipoamide branched chain transacylase E2), DHTKD1 (dehydrogenase E1 and transketolase domain containing 1), OAT (ornithine aminotransferase), and RALGAPB (Ral GTPase activating protein non-catalytic subunit beta) in liver and plasma exosomes. TYMP (thymidine phosphorylase) is the only protein showing coordinated upregulation in both muscle and plasma exosomes of large fish. Disclaimer: This diagram serves as a hypothetical and associative framework based on static multi-omics tissue correlations; the present descriptive data do not prove direct, active inter-organ transport or causal trafficking dynamics.

Based on proteomics data from the brain, liver, and muscle (data from our parallel study), further joint analysis was conducted, revealing several proteins that are significantly upregulated or downregulated in both the tissues and plasma exosomes of large and small fish. Among them, only TYMP (Thymidine Phosphorylase) was found to be significantly increased in both the muscle and plasma exosomes of large fish. GYG2 (Glycogenin 2), EBAG9 (Estrogen Receptor Binding Site Associated Antigen 9), RPZ (Rapunzel), and SLC2A2 (Solute Carrier Family 2 Member 2) were all significantly decreased in the brain and plasma exosomes of large fish. BHMT (Betaine—Homocysteine S-Methyltransferase), CLDN7B (Claudin 7b), DBT (Dihydrolipoamide Branched Chain Transacylase E2), DHTKD1 (Dehydrogenase E1 and Transketolase Domain Containing 1), OAT (Ornithine Aminotransferase), and RALGAPB (Ral GTPase Activating Protein Non-Catalytic Subunit Beta) were all significantly decreased in the liver and plasma exosomes of large fish. GYG2 was also significantly decreased in the muscle and plasma exosomes of large fish. Notably, most of the proteins identified here were significantly decreased in large fish and significantly increased in small fish, with the exception of TYMP, which was significantly increased in large fish. Furthermore, GYG2 was significantly decreased in the brain, plasma exosomes, and muscle of large fish, indicating a possible cross-tissue transmission pattern from brain to plasma to muscle. (Figure 8, Table S7).

## 3. Discussion

In this study, plasma exosomes were isolated and characterized from grass carp individuals exhibiting distinct growth phenotypes under high-density aquaculture conditions. Transmission electron microscopy, nanoparticle tracking analysis, and Western blot validation confirmed that the isolated vesicles exhibited typical exosomal characteristics, including cup-shaped morphology, a size range of 50–190 nm, and expression of the exosomal markers CD63, CD81, and TSG101. Our integrated multi-omics analysis identified 177 significantly differentially expressed miRNAs and 553 differentially enriched proteins in plasma exosomes between the large (fast-growing) and small (slow-growing) fish. Furthermore, we identified coordinated expression patterns of specific miRNAs and proteins across the brain, liver, and muscle tissues, providing correlative evidence of potential exosome-mediated cross-tissue communication in teleost fish.

### 3.1. Differential miRNA Expression Profiles Reflect Distinct Physiological States in Fast- and Slow-Growing Grass Carp

The miRNA profile analysis revealed distinct expression patterns between the two phenotypic groups. In large fish, miRNAs associated with immune modulation and inflammatory regulation, including members of the miR-142, miR-146, miR-150, miR-223, miR-462, miR-599, and miR-731 families, were significantly upregulated. Conversely, miRNAs associated with metabolism, skeletal and neural development, including miR-1, miR-10, miR-133, miR-143, miR-145, miR-196, miR-203, miR-204, miR-205, and miR-206 families, were predominantly downregulated in large fish.

From the perspective of evolutionary trade-offs, this systemic divergence in exosomal miRNA profiles under chronic high-density stress reveals a fundamental resource allocation strategy tailored to crowding. Intensive stocking induces subclinical inflammation in grass carp. The dramatic upregulation of immune-supportive and anti-inflammatory miRNAs in fast-growing individuals provides a robust physiological framework: these resilient fish may utilize exosomal signaling to buffer systemic inflammation, promote rapid leukocyte surveillance, and prevent the deleterious peripheral tissue damage typically triggered by chronic crowding. Concurrently, the synchronized downregulation of growth-suppressive miRNAs in the same large individuals likely lifts the brakes on peripheral protein synthesis and skeletal muscle hypertrophy. This metabolic uncoupling allows fast-growing fish to maintain active anabolic axes and tissue growth even under crowding stress, whereas slow-growing fish remain trapped in a growth-repressed state due to stress susceptibility.

This functional allocation is supported by established vertebrate literature. The upregulated cohorts (e.g., miR-142 [20], miR-146 [21], miR-223 [22]) are master regulators that restrain inflammatory signaling and modulate macrophage or B-cell activation under stress, while the miR-462/731 cluster is known to drive hematopoietic homeostasis in teleost [23]. Regarding the downregulated cluster, miR-1, miR-133, miR-203, and miR-206 have been characterized as critical negative regulators of muscle development, skeletal growth, and regeneration across various fish species [24,25,26]. Functional enrichment analysis of the target genes of the 177 differentially expressed miRNAs revealed results largely consistent with the previous literature review on miRNA functions. Most of the functions are primarily enriched in the developmental processes of the nervous system, skeletal system, and various tissues and organs, as well as in metabolic pathways involving substances such as proteins, amino acids, and nucleic acids. There are not many entries enriched in immunity and inflammation-related terms, but there are entries such as adaptive immune response and autophagosome maturation. The above analysis results suggest that plasma exosome miRNAs are closely linked to nutritional and developmental metabolic status, reflecting the diverging growth phenotypes of grass carp. The enrichment of neural development pathways is particularly intriguing, as it suggests potential links between neuroendocrine pathways and peripheral physiological states, as reflected by the exosomal signatures.

### 3.2. Proteomic Signatures Reveal Divergent Immune-Remodeling and Metabolic-Stress Phenotypes in Fast- and Slow-Growing Grass Carp

Proteomic analysis of plasma exosomes revealed complementary functional themes to those observed in the miRNA analysis. Proteins significantly upregulated in large fish were enriched in processes related to immune cell migration, inflammatory response, and cytoskeletal remodeling. Representative biological processes included locomotion, actin filament organization, leukocyte migration, chemotaxis, regulation of cell motility, and small GTPase-mediated signal transduction. Enrichment of cellular compartments such as basolateral/lateral plasma membrane, receptor complex, integrin complex, and cell leading edge further supports the involvement of these proteins in cell migration and tissue remodelling processes. Molecular functions, including GTPase regulator activity, actin binding, guanyl-nucleotide exchange factor (GEF) activity, and protein tyrosine kinase activity, suggest enhanced intracellular signaling capacity in fast-growing fish. From a standpoint of biological plausibility, the high enrichment of small GTPase-mediated signaling and cytoskeletal remodeling elements (such as actin-binding proteins and GEFs) in the exosome cargo of large fish presents a highly sophisticated adaptive mechanism to high-density aquaculture. Chronic crowding imposes continuous physical restriction and elevated encounter rates with pathogens. Small GTPases serve as master regulators that couple extracellular stress signals to rapid actin cytoskeleton dynamics, which are mandatory for both endothelial cell migration during angiogenesis and muscle fiber alignment during hypertrophy [27,28]. In teleosts, maintaining robust tissue repair and vascular expansion capabilities allows fast-growing individuals to dynamically adapt to high physical density, transforming environmental pressure into mechanical stimuli that promote anabolic growth rather than pathological injury. Concurrently, the exosome-mediated mobilization of leukocyte migration indicates that large fish possess superior immune surveillance. This allows for a swift, controlled inflammatory resolution at peripheral mucosal barriers, preventing localized infections from progressing into chronic, growth-depleting systemic inflammation [29]. Collectively, these findings indicate that fast-growing grass carp exhibit heightened immune and tissue repair activities, which may facilitate more efficient adaptation to the stressful high-density environment and promote anabolic processes conducive to rapid muscle hypertrophy. In contrast, proteins upregulated in small fish (or downregulated in large fish) were predominantly associated with energy metabolism, calcium ion regulation, muscle contraction, and endoplasmic reticulum (ER) stress response. Representative biological processes included calcium ion transport, glycolytic process, ADP metabolic process, response to ER stress, and muscle structure development. Enrichment of sarcoplasmic reticulum, sarcolemma, T-tubule, sarcomere, and Z disk as cellular compartments, along with calmodulin binding and calcium channel activity as molecular functions, highlights perturbations in muscle calcium homeostasis and energy production in slow-growing fish. The systemic upregulation of ER stress response proteins suggests that chronic crowding stress disproportionately breaches the protein-folding capacity of slow-growing individuals. In fish physiology, prolonged ER stress activates the evolutionarily conserved unfolded protein response (UPR) pathway [30,31]. While the UPR aims to restore homeostasis by clearing misfolded aggregates, it operates at an immense energetic cost and explicitly triggers the global attenuation of general protein translation to reduce the client load on the ER. Because muscle development in grass carp depends entirely on continuous, intensive protein synthesis and anabolism, this ER-stress-mediated translational arrest serves as a major molecular bottleneck [32]. These observations suggest that slow-growing fish experience greater metabolic stress and severely compromised muscle function compared to their fast-growing counterparts. The chronic stress inherent in high-density environments acts as a metabolic filter that drives extensive cellular distress in vulnerable individuals, causing energy reallocation toward basic survival and UPR activation rather than skeletal expansion, thereby strictly constraining growth potential [33].

### 3.3. Exosome-Mediated Cross-Tissue Communication Coordinates miRNA-Guided Gene Regulation in Muscle

One of the most significant findings of this study is the evidence for coordinated expression of specific miRNAs and proteins across brain, liver, and muscle tissues, supporting the potential involvement of exosomes in cross-tissue communication associated with growth variance. Through integrative analysis of miRNA sequencing and proteomic data from plasma exosomes and transcriptomic/proteomic data from growth-related tissues, we identified a subset of miRNAs and proteins that exhibited consistent expression patterns across multiple organs. Following qPCR validation, it was observed that the genes *alkbh5*, *dennd2db*, and *irf2bp2b* were significantly upregulated in the muscle of large fish. Predictive analyses indicate that these genes are regulated by bfl-miR-133, pma-miR-133a, and efu-miR-143, respectively. Concurrently, these three miRNAs were found to be significantly reduced in the liver and plasma exosomes of large fish, suggesting a coordinated expression network among liver, plasma exosomes, and muscle tissue. ALKBH5 is an m6A RNA demethylase that is upregulated in obese liver and coordinates hepatic glucose and lipid homeostasis by activating glucagon receptor- and mTORC1-dependent signaling [34]. Additionally, ALKBH5 was found to regulate mitochondrial ATP production in an m6A-dependent manner by stabilizing metabolic enzyme transcripts in hematopoiesis [35]. The increased *alkbh5* expression in large fish muscle may promote growth-associated transcriptome remodeling by reducing m6A modification on key muscle-related mRNAs, thereby enhancing the stability, processing, and translation of genes involved in myogenesis, protein anabolism, and muscle hypertrophy. DENND2DB, a DENN domain guanine nucleotide exchange factor that activates Rab9A/B, regulating endosome–Golgi trafficking and receptor recycling [36]. Its upregulation in the muscle of large fish may enhance membrane protein turnover and metabolic adaptation, thereby sustaining signaling and muscle hypertrophy during rapid growth. IRF2BP2B, a SUMO-dependent transcriptional repressor downstream of CCAAT enhancer binding protein alpha, suppresses the proviral integration oncogene to balance the fate of neutrophil and macrophage in zebrafish [37]. Its upregulation in the muscle of large fish may contribute to immune homeostasis, stress resilience, and tissue remodeling during growth.

### 3.4. Exosome-Mediated Transport of Growth-Regulatory Proteins Across Brain, Liver, and Muscle

**Figure 9:**
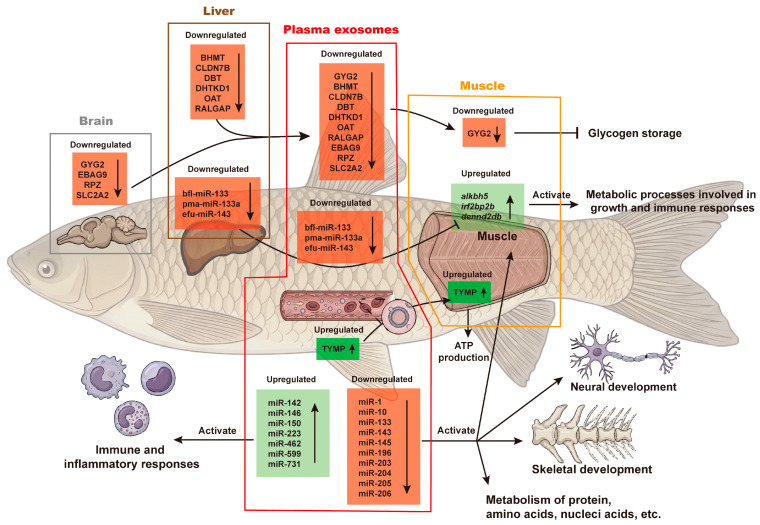
Schematic model of plasma exosomal molecular signatures associated with cross-tissue communication networks in large grass carp in high-density culture. Plasma exosomes from large grass carp carry distinct molecular cargoes that are associated with systemic signaling features between the brain, liver, and muscle. In large fish, plasma exosomes exhibit upregulation of immune-related miRNAs (miR-142, miR-146, miR-150, miR-223, miR-462, miR-599, and miR-731 families; green), which activate immune and inflammatory responses, and downregulation of growth-inhibitory miRNAs (miR-1, miR-10, miR-133, miR-143, miR-145, miR-196, miR-203, miR-204, miR-205, and miR-206 families; red), which promote neural development, skeletal development, and metabolism of proteins, amino acids, and nucleic acids. Notably, downregulation of bfl-miR-133, pma-miR-133a, and efu-miR-143 in liver and plasma exosomes (red) is associated with upregulation of their target genes (*alkbh5*, *irf2bp2b*, and *dennd2db*) in muscle (green arrows), thereby activating metabolic processes involved in growth and immune responses. At the protein level, TYMP is coordinately upregulated in both muscle and plasma exosomes (green), enhancing ATP production, whereas GYG2 is coordinately downregulated in brain, muscle, and plasma exosomes (red), reducing glycogen storage. Additionally, multiple proteins (BHMT, CLDN7B, DBT, DHTKD1, OAT, RALGAPB, EBAG9, RPZ, and SLC2A2) show coordinated downregulation in liver and/or brain together with plasma exosomes (red), reflecting metabolic reprogramming favoring rapid growth. Additionally, the downward arrows within the red boxes indicate downregulation, whereas the upward arrows within the green boxes indicate upregulation. The schematic illustrates how plasma exosomes reflect a systemic network integrating immune status, metabolic adaptation, and growth performance across multiple tissues under high-density aquaculture conditions.

At the protein level, GYG2 was consistently downregulated in the brain, plasma exosomes, and muscle of large fish, suggesting a coordinated reduction in glycogen synthesis capacity across tissues [38]. This indicates that fast-growing fish reduce the expression of proteins related to glycogen storage to meet high energy demands for growth. Conversely, TYMP is a key enzyme in nucleoside metabolism with additional roles in angiogenesis and tissue remodeling [39]. The upregulation of TYMP in the muscle and plasma exosomes of large fish may enhance mitochondrial stability and ATP production, while simultaneously promoting angiogenesis and tissue remodeling. This adaptive regulation likely supports efficient energy supply and vascularization required for rapid muscle hypertrophy. Additionally, BHMT, CLDN7B, DBT, DHTKD1, OAT, and RALGAPB were downregulated in both liver and plasma exosomes of large fish, while EBAG9, RPZ, and SLC2A2 were downregulated in both brain and plasma exosomes of large fish. BHMT catalyzes the use of betaine as a methyl donor to convert homocysteine into methionine. It is a key enzyme in hepatic methyl metabolism [40]. Its downregulation may indicate changes in DNA methylation patterns, promoting the expression of genes related to growth. CLDN7B is a member of the Claudin family, which is integral to the architecture of tight junctions. In hepatic tissue, Claudin proteins are vital for maintaining hepatocyte polarity, facilitating bile acid transport, and preserving barrier integrity [41]. The downregulation of CLDN7B in large fish may compromise the tight junctions between hepatocytes, thereby permitting the more facile transport of nutrients and metabolic factors across cell membranes or intercellularly. DBT serves as the central subunit of the pivotal enzyme responsible for catalyzing the degradation of branched-chain amino acids. Observations indicate a reduction in the levels of this protein in larger fish, suggesting a deceleration in the oxidative breakdown of branched-chain amino acids. This process aids in conserving essential amino acids for the synthesis of muscle proteins [42]. DHTKD1 is primarily responsible for catalyzing the oxidative decarboxylation of 2-oxoadipate within the degradation pathways of lysine and tryptophan [43]. The downregulation of this enzyme in large fish may suggest a deceleration in the catabolism of essential amino acids such as lysine, thereby ensuring their availability for protein synthesis. OAT catalyzes the transamination reaction between ornithine and α-ketoglutarate. The observed decrease in hepatic OAT in larger fish may represent a metabolic reprogramming strategy, in which reduced ornithine catabolism enhances ornithine availability for polyamine synthesis, thereby promoting cell proliferation and accelerated growth, while deprioritizing proline-dependent collagen synthesis [44]. RALGAPB functions as the non-catalytic subunit of the RalGAP complex. In conjunction with the catalytic subunit, it serves to inhibit the activity of RalA/RalB [45]. The downregulation of hepatic RALGAPB observed in large fish may reflect a release of inhibition on Ral GTPase activity, thereby accelerating mitotic progression and cellular proliferation. This regulatory shift could represent a metabolic and proliferative adaptation that facilitates rapid tissue expansion and overall growth. EBAG9 is an estrogen-responsive immunosuppressive protein that also regulates vesicular exocytosis via Snapin [46]. Its downregulation in the brain of large fish may enhance neurotransmitter release and reduce immune suppression, thereby supporting neuronal expansion and metabolic coordination for accelerated growth. RPZ is a protein whose gain-of-function mutation induces skeletal overgrowth in zebrafish, positioning it as a critical regulator of bone and tissue development [47]. The downregulation of RPZ in the brains of larger fish may serve as a compensatory mechanism to mitigate excessive structural growth, thereby reallocating resources towards neuronal connectivity and functional expansion. This adaptive regulation could facilitate the maintenance of proportional development while supporting accelerated growth. The *slc2a2* gene encodes the glucose transporter protein glucose transporter 2 (GLUT2), which is integral to the transmembrane transport and sensing of glucose. This protein plays a crucial role in regulating energy metabolism and maintaining blood glucose homeostasis within the body [48]. The downregulation of SLC2A2 in the brain of large fish may indicate a metabolic reprogramming that restricts cerebral glucose uptake, thereby reallocating energy substrates to peripheral growth tissues. This adaptive regulation potentially facilitates accelerated somatic growth. This coordinated expression pattern provides strong evidence for systemic molecular networks that co-vary with growth variation, highlighting the potential role of exosomes as message carriers (Figure 9).

### 3.5. Plasma Exosomal miRNAs and Proteins Linked to Immune Defense and Resource Allocation Under Stress

Integrating our multi-omics datasets reveals that the physiological adaptation and growth divergence of grass carp under chronic crowding stress are regulated by a systemic reallocation of biological resources. High-density crowding inevitably forces a physiological trade-off between host immune defense and somatic growth [49]. The findings of this study demonstrate that fast-growing grass carp exhibit a superior capacity to balance this trade-off via the differential expression of plasma exosomal cargo. At the miRNA level, fast-growing individuals maintain homeostatic balance by upregulating anti-inflammatory and immune-protective exosomal miRNAs (such as the miR-142 and miR-146 families) to mitigate crowding-induced systemic inflammation, while downregulating growth-inhibitory miRNAs (such as the miR-133 and miR-143 families). These miRNA dynamics correlate with the plasma proteomic profiles, where large fish show a significant enrichment in pathways associated with active leukocyte migration and cellular motility, whereas slow-growing fish exhibit signatures of persistent glycolytic stress and ER stress.

Crucially, these circulating macromolecular alterations reflect a highly coordinated, exosome-mediated cross-tissue communication network that regulates metabolic and neuroendocrine axes. The integration of parallel multi-tissue transcriptomic and proteomic data reveals that the systemic downregulation of circulating and hepatic bfl-miR-133, pma-miR-133a, and efu-miR-143 in large fish effectively removes post-transcriptional repression in peripheral tissues. This downregulation aligns with the significant muscular upregulation of target genes critical for myogenic remodeling, including *alkbh5*, *irf2bp2b*, and *dennd2db*. Simultaneously, at the proteomic level, plasma exosomes participate in long-range metabolic reprogramming. This is evidenced by the coordinated downregulation of GYG2 across the brain, circulation, and muscle of large fish to meet the high energy demands of rapid growth. Furthermore, the liver-to-exosome downregulation of amino acid catabolic enzymes (DBT, DHTKD1, OAT, and BHMT) in large fish suggests a mechanism to conserve nitrogen building blocks for protein anabolism. Conversely, the synchronized upregulation of TYMP in both the muscle and plasma exosomes of fast-growing fish supports enhanced angiogenic adaptation and ATP efficiency required for skeletal muscle hypertrophy under crowded conditions. In summary, these interconnected multi-omics signatures demonstrate that plasma exosomal profiles are closely associated with immune resilience, metabolic plasticity, and cross-tissue communication, thereby reflecting the physiological mechanisms linked to growth divergence in teleosts under aquaculture crowding stress.

### 3.6. Study Limitations, Future Directions, and Translational Potential of Exosome-Based Growth Biomarkers

The identification of cross-tissue communication via plasma exosomes in grass carp aligns with accumulating evidence from mammalian and other vertebrate models, where exosomes have been shown to mediate interorgan communication in metabolic regulation, immune responses, and tissue repair [50]. According to our current knowledge, this study represents one of the first comprehensive investigations into exosome-mediated cross-tissue communication in teleost fish, particularly regarding growth regulation. These findings provide new insights into the molecular mechanisms underlying growth variation in aquaculture species and highlight the potential of targeting exosome-mediated signaling pathways for improving growth performance. Several limitations of this study should be acknowledged. Although our exosome multi-omics profiling and qPCR validations utilized a sample-pooling strategy to secure sufficient input material, the final evaluation was constrained to *n* = 3 biological replicates per cohort. This configuration introduces a degree of technical pseudoreplication that may mask individual-level biological variability. While this sample size provides adequate statistical power to discern major adaptive molecular axes under intensive crowding stress, future investigations expanding the cohort scale and incorporating individual un-pooled functional validation will be essential to comprehensively map out the finer layers of biological variability and direct mechanistic causality in teleost exosome signaling. Additionally, the genetic sex of the grass carp was not determined in this study, and circulating primary stress parameters (such as plasma cortisol or glucose levels) were not monitored. Although sexual growth dimorphism is documented in certain adult teleosts, empirical studies in pond-cultured grass carp establish that body weight growth curves between females and males remain statistically indistinguishable prior to 16 months of age, with full sexual maturity achieved only at 4 to 5 years. Given that our fish were sampled at a highly immature juvenile stage (approximately 10 months old), sex-specific endocrine or metabolic axes were physiologically dormant and unlikely to confound the growth divergence observed here. Nevertheless, the absence of longitudinal cortisol mapping limits our capacity to directly couple exosomal dynamics with classical neuroendocrine stress axes. Importantly, a key limitation of the present study is its correlational and associative design. Based on the current multi-omics data, we cannot definitively determine whether the observed differences in exosomal miRNAs and proteins are the driving causes or merely the physiological consequences of growth divergence. It is highly possible that the distinct exosomal profiles in large and small fish represent a downstream metabolic byproduct of their differential growth rates and energy states under high-density stress. Nonetheless, even as a consequence, these coordinated molecular signatures remain robust, non-invasive biomarkers that reflect the physiological and growth status of teleosts. Further mechanistic investigations, including the application of synthetic miRNA mimics in vitro and exosome delivery approaches in vivo, are essential to clarify the precise cause-and-consequence relationships underlying these pathways. Future studies should aim to increase sample sizes, include validation cohorts, and employ functional assays to test the effects of identified exosomal miRNAs and proteins on growth-related cell types (e.g., myocytes, hepatocytes). Techniques such as exosome treatment of cultured cells, miRNA mimics/inhibitors transfection, and protein overexpression/knockdown studies could provide mechanistic insights into how these exosomal cargoes influence growth processes. From a practical aquaculture perspective, the identification of plasma exosome miRNAs and proteins that distinguish fast- and slow-growing individuals has significant implications for breeding programs. These molecular signatures could serve as early, non-invasive biomarkers for predicting growth potential, potentially reducing the time and cost associated with traditional selective breeding methods. Exosome-based biomarker screening enables the early identification of fish suitable for high-density farming, facilitating selection at juvenile stages and thereby significantly shortening breeding cycles. Furthermore, understanding the molecular basis of growth variation could inform targeted interventions, such as nutritional supplementation or environmental management strategies, to mitigate growth disparities under high-density conditions.

## 4. Materials and Methods

### 4.1. Experimental Design and Fish Husbandry

The experiment involving high-density grass carp (*Ctenopharyngodon idella*) was conducted over a period of nine months, from August 2024 to May 2025, at Guangdong Chengyi Industrial Group Co., Ltd., Guangzhou, China. Initially, 14200 fish with an average initial body weight of 19 g were stocked on 5 August 2024. During the culture period, fish were fed twice daily at a feeding rate of 2% of their body weight. Throughout the trial, stocking densities and dissolved oxygen (DO) levels were strictly monitored. The stocking density reached a maximum of 32,430 kg/ha (2162 kg/mu) in late September 2024, with DO levels ranging from 1 to 5 mg/L. In contrast, the typical density during this period was approximately 13,110–16,605 kg/ha (874–1107 kg/mu), with DO levels between 3 and 7 mg/L. Therefore, the stocking density observed at peak in the study was approximately 2.0 to 2.5 times greater than the normal density. During the husbandry, the stocking density was adjusted by reducing the number of fish to maintain optimal growth conditions. Such a high-intensity environment was maintained for 9 months to induce chronic physiological adaptations. At the conclusion of the 9-month trial in May 2025, the final population stood at about 9400 fish. At the end of the 9-month experimental period, a subgroup of 25 fish was randomly caught from the pond population. Based on their body weights, 6 large individuals (the largest within the subgroup) and 15 small individuals (the smallest within the subgroup) were subsequently selected for tissue sampling. Prior to sampling, fish were anesthetized, and blood was collected from the caudal vein to obtain plasma for exosome extraction. Subsequently, the brain, liver, and muscle tissues were rapidly excised, snap-frozen in liquid nitrogen, and stored at −80 °C for further molecular analysis.

### 4.2. Plasma Exosome Isolation and Characterization

#### 4.2.1. Plasma Exosome Isolation

We combined the plasma from six large fish in pairs to create three biological replicates for the large fish group (L). The plasma from fifteen small fish was pooled every five individuals in each sample, forming three samples for the small fish group (S). This approach was used to increase the yield of plasma exosomes from each sample to meet the requirements for subsequent exosome characterization, miRNA sequencing, and proteomic sequencing. While this sample pooling strategy was necessary to ensure sufficient exosome yields, we acknowledge that it presents a limitation regarding pseudoreplication, which may reduce the ability to assess individual biological variation. Consequently, the key regulatory networks identified here warrant validation using separate, individual biological specimens in future studies. Exosomes were isolated from plasma using a differential ultracentrifugation (UC) method specifically adapted for viscous fluids, with modifications tailored to this study [51]. The process began by diluting plasma samples with an equal volume of PBS, followed by centrifugation at 2000× *g* for 30 min. The supernatant was carefully collected to avoid disturbing the sediment and transferred to fresh tubes for a subsequent centrifugation at 12,000× *g* for 45 min. This supernatant was then transferred to an ultracentrifuge tube (361625, Beckman Coulter) with care to prevent contamination, and ultracentrifugation was performed at 120,000× *g* for 2 h (Optima XE-100, Beckman Coulter, Indianapolis, IN, USA). The resulting precipitate was resuspended in PBS. The filtrate was subjected to a second ultracentrifugation at 120,000× *g* for 70 min. The resulting pellet was resuspended in PBS and underwent a third ultracentrifugation at 120,000× *g* for 70 min. Finally, the pellet was resuspended in a moderate amount of PBS and stored at −80 °C for subsequent exosome analysis. All centrifugation steps were conducted at 4 °C.

#### 4.2.2. Western Blotting

The samples were lysed using RIPA lysis buffer (P0013K, Beyotime, Shanghai, China), and the total protein concentration was quantified employing the bicinchoninic acid (BCA) assay kit, following the manufacturer’s instructions (BL1054A, Biosharp, Hefei, China). Subsequently, 20 μg of protein was subjected to separation via sodium dodecyl sulfate polyacrylamide gel electrophoresis (SDS-PAGE) and transferred onto polyvinylidene fluoride (PVDF) membranes (Millipore, China, Burlington, MA, USA). The membranes were blocked with blocking buffer (PR20034, Proteintech, Rosemont, IL, USA) for 2 h at room temperature and then incubated with specific primary antibodies for 16 h at 4 °C. The primary antibodies utilized in this study included CD63 (25682-1-AP, 1:1000 dilution, Proteintech), CD81 (27855-1-AP, 1:3000 dilution, Proteintech), and TSG101 (84334-4-RR, 1:3000 dilution, Proteintech). Following this, the membranes were washed three times with TBST (TBS containing 0.1% Tween-20) and incubated with a Multi-rAb^®^ (Rosemont, IL, USA) HRP-goat anti-rabbit recombinant secondary antibody (RGAR001, 1:10,000 dilution, Abcam, Cambridge, UK) for 2 h at room temperature. Protein visualization was achieved using chemiluminescent reagents (AR1171S, Boster, Pleasanton, CA, USA) after washing with TBST by a chemiluminescence imaging system (e-BLOT, Yibote Life Sciences, Wuhan, China).

#### 4.2.3. Transmission Electron Microscopy

Exosomes were immobilized on a 200-mesh carbon/formvar-coated grid by inverting the grid onto an exosome droplet (10 μL) for a duration of 10 min. Subsequently, the grid was transferred to a droplet of distilled water for 1 min, followed by immersion in 2.5% glutaraldehyde for 10 min. Post-fixation, the absorbed exosomes underwent negative staining with 2% uranyl acetate for 1 min. The grid was then examined using a transmission electron microscope operated at 120 kV (H-7650, Hitachi, Tokyo, Japan).

#### 4.2.4. Nano-Flow Cytometry (nFCM)

The particle size distribution and concentration of exosomes were evaluated utilizing a nano flow cytometer (Flow NanoAnalyzer, NanoFCM, Xiamen, China). The sample was diluted with phosphate-buffered saline (PBS) to achieve a particle concentration of 10^8^/mL for detection purposes. The concentration measurement involved detecting the number of fluorescent silica nanoparticles (250 nm) with a known concentration over a specified time interval. By calculating the sample flow rate and integrating it with the particle count under identical injection pressure conditions, the particle concentration of the sample was rapidly determined. The particle size distribution was established using standard silica beads (68 nm, 91 nm, 113 nm, 155 nm) to construct a standard working curve correlating scattered light intensity with particle size. The scattering intensity of the test sample under identical conditions was then converted into particle size information, thereby yielding the particle size distribution of the sample under investigation.

### 4.3. Exosome miRNA Extraction, Library Construction, Sequencing, and Analysis

Total exosomal RNA was extracted using the Exosome RNA Purification Kit (Simgen, China, Hangzhou, China) according to the manufacturer’s instructions. For sequencing library preparation, 3 ng of total RNA was used as input using the QIAseq miRNA Library Kit (Qiagen, Hilden, Germany) following the manufacturer’s protocols, which incorporates unique molecular identifiers (UMIs) to track individual RNA molecules. The constructed libraries were sequenced on an Illumina NovaSeq X Plus platform (Illumina, San Diego, CA, USA). Raw data in fastq format were initially processed using an in-house small-RNA analysis pipeline (v2.5.5) with UMI support. Clean data were obtained by removing reads with adapters, reads containing >10% ambiguous bases (poly-N), and low-quality reads from raw data. Reads were trimmed and retained only if within the configured small-RNA length range (between 15 nt and 35 nt). Q20, Q30, GC-content, and sequence duplication level of clean data were calculated. All downstream analyses used high-quality clean data. Bowtie (v2.5.4) tools were used for clean read alignment with the Silva, GtRNAdb, Rfam, and Repbase databases to filter rRNA, tRNA, snRNA, snoRNA, other ncRNA, and repeats. Remaining reads detected known and novel miRNAs by comparing the genome and miRbase [52]. Randfold (v2.0) tools predicted novel miRNA secondary structures. Gene function annotation was performed using the Nr, Pfam, KOG/COG, Swiss-Prot, KEGG, and GO databases. The miRNA expression levels were estimated: 1. sRNAs were mapped to the precursor sequences. 2. miRNA read count from the mapping results. 3. Reads with the same UMI were unique to one read. Differential expression analysis of conditions/groups/samples was performed using the edgeR R package (3.12.1), which provides statistical routines for differential expression in digital miRNA data based on Poisson distribution and Empirical Bayes [53]. *p* values were adjusted using Benjamini and Hochberg’s method for the false discovery rate. Utilizing the grass carp (*Ctenopharyngodon idella*) reference genome GCF_019924925.1 for alignment, miRNA identification was conducted on the clean reads from the six samples (large fish group: L1–L3; small fish group: S1–S3). miRanda (v3.3a) and RNAhybrid (v2.1.1) software were utilized to predict the target genes of miRNAs. GO enrichment analysis of differentially expressed genes (DEGs) was performed using widely adopted R packages specialized for GO analysis [54]. KEGG pathway analysis was conducted to interpret the biological functions and pathways associated with the DEGs, utilizing KEGG as a comprehensive resource for molecular-level functional annotation [55]. To enhance statistical transparency and minimize the background noise of low-expressed sequences for downstream biomarker selection, an additional stringent abundance filter was applied following edgeR profiling: only miRNAs with a raw expression value exceeding 100 in at least one sample were retained, resulting in a high-confidence core set of 177 candidate miRNAs for target gene prediction and tissue co-variance verification.

### 4.4. Exosome Proteomics Sequencing and Analysis

Exosomes were isolated and placed in a tube cooled with liquid nitrogen. They were then disrupted using an ice water bath and Ultrasonic Processors, with the process involving intermittent pauses every 30 s for 10 s, continuing for a total duration of 10 min. Following centrifugation at 14,000× *g* for 20 min, the supernatant was collected. The protein concentration was quantified using the BCA method, and the remaining sample was stored at −80 °C. 10 μg of protein from each sample was processed using SISPROT, a technology designed to streamline protein handling. SISPROT (BayOmics, Guangzhou, China) facilitates the integration of protein processing in a single step by employing a spin tip equipped with a C18 membrane and SCX/SAX beads. Peptides were preserved at −80 °C. Two solutions were formulated: Solution A, consisting of 100% water and 0.1% formic acid, and Solution B, comprising 80% acetonitrile and 0.1% formic acid. Peptides were reconstituted in 10 µL of Solution A, subjected to centrifugation at 14,000× *g* for 20 min at 4 °C, and 300 ng of the supernatant was utilized for LC-MS detection. The analysis was conducted using a Vanquish Neo UHPLC system with a C18 pre-column maintained at 50 °C and a C18 analytical column. A Thermo Orbitrap Astral mass spectrometer (Waltham, MA, USA), equipped with an Easy-spray ion source, was employed. The ion spray voltage was set at 1.9 kV, and the ion transfer tube was maintained at 290 °C. Mass spectrometry was performed in DIA mode, covering a range of *m*/*z* 380–980. The primary MS resolution was 240,000 (200 *m*/*z*), with an AGC target of 500%, a parent ion window of 2-Th, 300 DIA windows, an NCE of 25%, a secondary m/z range of 150 to 2000, a sub-ion resolution of 80,000, and a maximum injection time of 3 ms. All RAW files were checked using DIA-NN (v1.8.1). MS2 spectra were compared to the *Ctenopharyngodon idella* reference database retrieved from NCBI (GCF_019924925.1_HZGC01).

Protein samples showed distinct missing data patterns across the six samples. To prevent mass spectrometry artifacts from biasing the downstream analysis, proteins with substantial missing values (defined as a total valid value ≤ 1 in each experimental group) were strictly excluded, removing 202 low-confidence proteins and retaining a stable dataset of 4001 proteins. For remaining patterns with partial data, missing values were estimated using k-Nearest Neighbors (KNN), while cases with complete absence in one group were evaluated using Fisher’s exact test. After data processing, differential expression analysis was performed using the limma package (v3.46.0) to calculate *p*-values, log2 fold changes (log2FC), and false discovery rates (FDR). To ensure maximum identification confidence and focus on robust physiological alterations under long-term high-density aquaculture stress, proteins were considered significantly differentially expressed only if they exhibited a False Discovery Rate (FDR) < 0.05 and a strict absolute fold change limit of |FC| > 2.0. GO and KEGG pathway enrichment analyses of the exosome differentially expressed proteins (DEPs) were conducted using the same methodology as applied to the DEGs in Section 4.3.

### 4.5. Quantitative Real-Time PCR

Gene expression analysis in muscle was performed using quantitative reverse transcription polymerase chain reaction (qRT-PCR). RNA was extracted from these tissues employing the RNA isolation kit (DP501, Tiangen, Beijing, China). Complementary DNA (cDNA) synthesis for messenger RNAs (mRNAs) was conducted using the reverse transcription kit (AG11728, Accurate Biotechnology, Changsha, China). The qPCR was carried out on 384-well plates utilizing the LightCycler 480 II qRT-PCR system (Roche Diagnostics, Basel, Switzerland). For the analysis of gene expression, the qPCR PreMix kit (AG11701, Accurate Biotechnology) was employed, following a protocol of pre-denaturation at 95 °C for 30 s, followed by 40 cycles of denaturation at 95 °C for 5 s, and annealing and extension at 60 °C for 30 s. A melting curve analysis was performed at the end of each program. Each PCR assay included duplicate samples and negative controls. β-actin was used as the normalization control for gene expression. The relative expression levels were calculated using the E-Method from the LightCycler 480 software (version SW 1.5; Roche Diagnostics). The primers used for RT-qPCR are specified in Table 2.

## 5. Conclusions

In conclusion, this study provides a comprehensive characterization of plasma exosome miRNA and protein profiles from grass carp with distinct growth phenotypes under high-density aquaculture conditions. Our findings highlight the importance of exosome-mediated cross-tissue communication in regulating growth and adaptation to environmental stress. The upregulation of immune-related miRNAs and proteins, along with the downregulation of metabolic stress markers, in fast-growing fish suggests that efficient systemic signaling and stress adaptation are key determinants of superior growth performance. The identified exosomal signatures have potential applications as biomarkers for growth selection and may guide future research aimed at improving growth efficiency in aquaculture species through targeted manipulation of cross-tissue communication pathways.

## Figures and Tables

**Table 1:** miRNA–target gene pairs showing coordinated expression between plasma exosomes and growth-related tissues.

miRNAs	miRNA Sequences	miRNA Regulation	Symbol of Target Genes	Name of Target Genes	Target Gene Regulation
dre-miR-146b	TGAGAACTGAATTCCAAGGGTG	Up regulated in brain and plasma exosome of large fish	*rars2*	arginyl-tRNA synthetase 2, mitochondrial	Down regulated in liver of large fish
		Up regulated in brain and plasma exosome of large fish	*ttc19*	tetratricopeptide repeat domain 19	Down regulated in liver of large fish
bfl-miR-133	TGGTCCCCTTCAACCAGCTGTA	Down regulated in liver and plasma exosome of large fish	*cped1*	cadherin like and PC-esterase domain containing 1	Up regulated in muscle of large fish
		Down regulated in liver and plasma exosome of large fish	*alkbh5*	alkB homolog 5, RNA demethylase	Up regulated in muscle of large fish
		Down regulated in liver and plasma exosome of large fish	*saa*	serum amyloid A-1 protein	Up regulated in muscle and liver of large fish
dme-miR-133-3p	TTGGTCCCCTTCAACCAGCTGT	Down regulated in liver and plasma exosome of large fish	*cbln14*	cerebellin 14	Up regulated in muscle of large fish
		Down regulated in liver and plasma exosome of large fish	*cped1*	cadherin like and PC-esterase domain containing 1	Up regulated in muscle of large fish
efu-miR-133-3p	TTTGGTCCCCTTCAACCAGCTGTA	Down regulated in liver and plasma exosome of large fish	*cped1*	cadherin like and PC-esterase domain containing 1	Up regulated in muscle of large fish
		Down regulated in liver and plasma exosome of large fish	*egln1a*	egl-9 family hypoxia-inducible factor 1a	Up regulated in muscle of large fish
ola-miR-133-3p	TGGTCCCCTTCAACCAGC	Down regulated in liver and plasma exosome of large fish	*saa*	serum amyloid A-1 protein	Up regulated in muscle and liver of large fish
		Down regulated in liver and plasma exosome of large fish	*cbln14*	cerebellin 14	Up regulated in muscle of large fish
		Down regulated in liver and plasma exosome of large fish	*cped1*	cadherin like and PC-esterase domain containing 1	Up regulated in muscle of large fish
pma-miR-133a	TTGGTCCCCTTCAACCAGCTTG	Down regulated in liver and plasma exosome of large fish	*irf2bp2b*	interferon regulatory factor 2 binding protein 2b	Up regulated in muscle of large fish
		Down regulated in liver and plasma exosome of large fish	*cped1*	cadherin like and PC-esterase domain containing 1	Up regulated in muscle of large fish
		Down regulated in liver and plasma exosome of large fish	*cbln14*	cerebellin 14	Up regulated in muscle of large fish
ppy-miR-133a	TTGGTCCCCTTCAACCAGCTG	Down regulated in liver and plasma exosome of large fish	*cped1*	cadherin like and PC-esterase domain containing 1	Up regulated in muscle of large fish
		Down regulated in liver and plasma exosome of large fish	*cbln14*	cerebellin 14	Up regulated in muscle of large fish
sha-miR-133a	TTTGGTCCCCTTCAACCAG	Down regulated in liver and plasma exosome of large fish	*cped1*	cadherin like and PC-esterase domain containing 1	Up regulated in muscle of large fish
cpi-miR-133a-3p	TTTGGTCCCCTTCAACCAGCTGT	Down regulated in liver and plasma exosome of large fish	*cped1*	cadherin like and PC-esterase domain containing 1	Up regulated in muscle of large fish
gga-miR-133b	TTGGTCCCCTTCAACCAGCTA	Down regulated in liver and plasma exosome of large fish	*cbln14*	cerebellin 14	Up regulated in muscle of large fish
		Down regulated in liver and plasma exosome of large fish	*cped1*	cadherin like and PC-esterase domain containing 1	Up regulated in muscle of large fish
pma-miR-133b-3p	TTTGGTCCCCTTCAACCAGCTC	Down regulated in liver and plasma exosome of large fish	*egln1a*	egl-9 family hypoxia-inducible factor 1a	Up regulated in muscle of large fish
gga-miR-133c-3p	TTGGTCCCCTTCAACCAGCTGC	Down regulated in liver and plasma exosome of large fish	*wdr77*	WD repeat domain 77	Up regulated in muscle and liver of large fish
		Down regulated in liver and plasma exosome of large fish	*cped1*	cadherin like and PC-esterase domain containing 1	Up regulated in muscle of large fish
		Down regulated in liver and plasma exosome of large fish	*cbln14*	cerebellin 14	Up regulated in muscle of large fish
efu-miR-143	GTCTGAGATGAAGCACTGTAGCTC	Down regulated in liver and plasma exosome of large fish	*wu:fb55g09*	uncharacterized	Up regulated in muscle of large fish
		Down regulated in liver and plasma exosome of large fish	*dennd2db*	DENN/MADD domain containing 2Db	Up regulated in muscle of large fish
		Down regulated in liver and plasma exosome of large fish	*LOC127509884*	GTPase IMAP family member 8-like	Up regulated in muscle of large fish
xtr-miR-133d	TTGGTCCCCTTCAACCAGCCGC	Down regulated in muscle and plasma exosome of large fish	*cped1*	cadherin like and PC-esterase domain containing 1	Up regulated in muscle of large fish
		Down regulated in muscle and plasma exosome of large fish	*cbln14*	cerebellin 14	Up regulated in muscle of large fish

**Table 2:** Sequences of the primers for quantitative real-time PCR.

Gene	Forward Primers	Reverse Primers
*cped1*	ACCCTCCAATGGTGCTGATG	GCTCGTCCAGGTGAATGTGA
*alkbh5*	GATGCCCCTCGCTTAGACTC	AAATACGAGGCCTGTGTGCA
*saa*	GGACTGACCTGAGCAAACCA	CTTGGATGGCTTCTCCTGGG
*egln1a*	GCCGGATCTCCTTCTACTGC	GCCTCTCTGCAGGTGAGTTT
*irf2bp2b*	GCTGGGGACGTAAAGGTGAA	TCCGTTAGTTCGCGTTTGGA
*wdr77*	GGATCTGAACCAGGAGAGCG	TCTGCAGGAGAACACGACAC
*dennd2db*	TCGCACCTGAACTTCGTCAA	GGGCTGCATTTCCTCTTCCT
*LOC127509884*	CACTGATGGTGTGAGGACCC	CGAGCATGTTTAGGCCCTCA
*β-actin*	CTGGCCCCTAGCACAATGAA	GCCATGCCAATGTTGTCGTT

## Data Availability

The raw miRNA-seq data have been deposited in the NCBI Gene Expression Omnibus (GEO) under accession number GSE336504. The proteomics data have been submitted to the ProteomeXchange Consortium via the PRIDE partner repository under the accession number PXD080172.

## Supplementary Materials

The following supporting information can be downloaded at: https://www.mdpi.com/article/10.3390/ijms27136059/s1.
